# An In Vitro Investigation of the Antiproliferative and Antimetastatic Effects of Levosimendan: Potential Drug Repurposing for Cervical Cancer

**DOI:** 10.3390/cimb46070391

**Published:** 2024-06-27

**Authors:** Zsuzsanna Schelz, Hiba F. Muddather, Fatemeh Sheihaki Jaski, Noémi Bózsity, István Zupkó

**Affiliations:** Institute of Pharmacodynamics and Biopharmacy, Faculty of Pharmacy, University of Szeged, Eötvös u. 6, H-6720 Szeged, Hungary; schelz.zsuzsanna@szte.hu (Z.S.); hiba.161991@hotmail.com (H.F.M.); jaski.sheihaki.fatemeh@gmail.com (F.S.J.); bozsity-farago.noemi@szte.hu (N.B.)

**Keywords:** levosimendan, cervical cancer, anticancer, antimetastatic, repurposing

## Abstract

Cervical cancer presents a significant challenge to the global health of women. Despite substantial advances in human papillomavirus (HPV)-related cervical cancer vaccines, non-HPV-related cervical cancer is still waiting novel therapeutic options. Drug repurposing has provided a promising approach to improve cancer therapy in recent years. Our study aimed to explore the potential in vitro antineoplastic effects of levosimendan on cervical cancer cells. The antiproliferative effects of levosimendan were investigated on cervical cancer cells using a standard MTT assay. Fluorescent double staining was performed to identify its ability to induce apoptosis and necrosis. The possible mechanism of action of levosimendan was explored using cell-cycle analysis. Furthermore, antimetastatic effects were investigated using a wound-healing assay and a Boyden chamber assay. Our results revealed that levosimendan exhibited the highest growth-inhibitory effect in the HPV-negative C33A cell line. However, the effects were modest compared to the standard agent, cisplatin. Cell-cycle analysis detected that levosimendan can induce cell-cycle arrest in C33A cells by increasing the G1 and G2/M phases, decreasing the S phase, and enhancing the hypodiploid subG1 population. Levosimendan inhibited cell migration and invasion in a concentration-dependent manner. As levosimendan showed antimetastatic efficacy, it could be considered for repurposing to contribute to overcoming resistance to therapy in cervical cancer.

## 1. Introduction

Cancer is a major global health burden, escalating in incidence and fatality [1]. Among women, cervical cancer is the fourth most frequently diagnosed cancer and the fourth leading cause of cancer-related death in 2020, with a global burden estimate of 604,000 new cases and 342,000 deaths. Cervical cancer is the most commonly diagnosed cancer in women in 23 countries and the leading cause of cancer-related death in 36 countries [2]. The highest estimated age-standardized incidence of cervical cancer is 40.1 per 100,000 women (observed in eastern Africa) [2]. Although cervical cancer incidence has been decreasing in developed countries, developing countries have encountered an increase in overall cancer incidence and mortality [3,4]. Socioeconomic differences and numerous factors, such as low access to screening programs, inadequate preventive approaches, unsuccessful treatment regimens, and poor health conditions, are attributed to geographical discrepancies in the incidence and mortality of cervical cancer [5,6].

Cervical cancer is predominantly correlated with human papillomavirus (HPV) infection and comprises 84% of all HPV-related cancer lesions [7]. The oncogenesis of numerous low- and high-risk strains of HPV has been identified and related to the most common types of cervical cancer. Of these, two types of HPV, HPV16 and HPV18, have been identified as the most prevalent and responsible for 70% of cervical cancers and precancerous cervical lesions [8,9]. The WHO recommends routine HPV vaccination for girls aged 9–13 years of age with a two-dose vaccination [2].

Cervical cancer is a largely preventable disease due to the effectiveness of preventive measures such as the HPV vaccine and screening. However, despite advances in screening and the high efficacy and safety of HPV vaccines, national HPV vaccination programs are still lacking and vaccination coverage rates are still minimal in most regions of the world [10,11,12,13]. Nonetheless, less than 10% of cases of invasive cervical cancer are classified as truly HPV-negative [14,15,16,17]. Patients with HPV-negative cervical cancer show a significantly impaired prognosis due to the advanced stage of the International Federation of Gynecology and Obstetrics (FIGO), with a higher prevalence of lymphatic invasion at diagnosis than HPV-positive cervical cancer [18,19,20,21]. More attention must be paid to HPV-negative cervical cancer management plans to mitigate its prognosis.

Cancer-related mortality remains a global health challenge regardless of the progress in treatments [22,23]; one of the causes could be attributed to acquired resistance to chemotherapy or the intolerability of the side effects of the currently available chemotherapy, highlighting the urgent need for safer agents with anticancer properties [24,25].

Drug repurposing, also known as repositioning, is the discovery of new applications for drugs that have already been approved and marketed [26]. This strategy considerably reduces the cost and research time compared to traditional drug discovery and development, as the drug pharmacokinetics, safety profile, and tolerability have already been assessed [27,28]. Thalidomide is an example of a successfully repurposed drug used in the treatment of cancer which was originally approved as a sedative that can treat nausea during pregnancy and is currently used in the management of multiple myeloma [29]. Numerous studies have reported the possibility of repurposing cardiovascular drugs in cancer management. Examples of these drugs include statins [30], aspirin [31], cardiac glycosides [32], beta-blockers [33], angiotensin-converting enzyme (ACE) inhibitors, and angiotensin-receptor blockers (ARBs) [34,35]. Levosimendan is an approved cardiovascular drug for the treatment of heart failure that exerts a positive inotropic effect by sensitization to calcium by binding with cardiac troponin C, leading to elevated cardiac contractility without increasing the energy and oxygen demand [36,37]. Furthermore, it exerts its inotropic action through vasodilatory effects [38,39]. Moreover, levosimendan has antiarrhythmic effects in acute myocardial infarction [40,41] and improves ventricular contractile function [42]. The vasodilatory effects of levosimendan have been attributed to its ability to activate ATP-sensitive potassium channels [43,44]. Additionally, levosimendan has been categorized as a potent inhibitor of phosphodiesterase (PDE) III that does not affect PDE IV at lower drug concentrations [45,46], and part of its action has been explained by the inhibition of PDE and subsequent elevation of intracellular cyclic adenosine monophosphate (cAMP) [47]. Nitric oxide (NO) production stimulated by levosimendan further improves its vasorelaxation effects [48]. 

Although accumulating evidence suggests the potential anticancer effects of levosimendan [49], scarce data on the potential anticancer mechanisms of this drug are published [50]. Therefore, according to our knowledge, we conducted the first study to assess the antitumor and antimetastatic properties of levosimendan on cervical cancer cells and approach its possible mechanism of action.

## 2. Materials and Methods

### 2.1. Chemicals

Levosimendan was purchased from Sigma-Aldrich Ltd. (Budapest, Hungary). Stock solutions of levosimendan were made in dimethylsulfoxide (DMSO) at 100 mM concentration and then diluted with culture medium to the required concentrations before use. Unless specified otherwise, the chemicals and kits used for the experiments were purchased from Merck Life Science Ltd. (Budapest, Hungary). The media were obtained from Capricorn Scientific GmbH (Ebsdorfergrund, Germany), and the media supplements were purchased from Lonza Group Ltd. (Basel, Switzerland). 

### 2.2. Cell Lines and Culture

Human-derived cervical cancer cell line SiHa (HPV16-positive squamous carcinoma) and C33A (HPV-negative carcinoma) cervical cell line were acquired from the American Tissue Culture Collection (ATCC, Manassas, VA, USA). The HeLa (HPV18-positive squamous carcinoma) cell line was obtained from the European Collection of Cell Cultures (Salisbury, UK). Cells were grown in a minimal essential medium (MEM) supplemented with 10% heat-inactivated fetal bovine serum (FBS), 1% non-essential amino acids, and 1% antibiotic-antimycotic mixture at 37 °C in humidified atmospheric air with 5% carbon dioxide (CO_2_). The non-cancerous human lung fibroblast cell line (MRC-5) was a generous gift from Professor Mónika Kiricsi (University of Szeged, Hungary) that was cultured in low-glucose DMEM accompanied with 20% FBS, 1% antibiotic-antimycotic mixture, and 4 mM L-glutamine at the previously mentioned cell culture conditions.

### 2.3. Antiproliferative MTT Assay

The growth-inhibitory effect of levosimendan was determined by a standard colorimetric MTT assay on three cervical cancer cell lines with diverse HPV statuses: HeLa, SiHa, and C33A cells. Non-cancerous MRC-5 was also used to assess cancer selectivity [51]. Briefly, SiHa and HeLa cells were seeded in 96-well microplates at a density of 5000 cells/well, while C33A and MRC-5 cells were seeded at 10,000 cells/well and incubated overnight. Cells were then treated with different concentrations of levosimendan (0.1–300 μM). Untreated wells containing cells were used as controls, and cisplatin was utilized as a positive control. After incubation for 72 h at 37 °C, 5 mg/mL MTT (3-(4,5-dimethyl- azole-2-yl)-2,5-diphenyl-2H-tetrazolium bromide) in phosphate buffer solution was added, followed by another 4 h of incubation. The water-insoluble precipitated purple formazan crystals were dissolved in DMSO, and the absorbances were measured with a microplate reader (BMG Labtech, Ortenberg, Germany) at 545 nm. The GraphPad Prism v 9.0 software (GraphPad Software, San Diego, CA, USA) fitted normalized dose–response curve data, and IC_50_ values were calculated. The experiments were performed with five parallel wells for each experimental condition and repeated twice.

### 2.4. Hoechst 33258–Propidium Iodide Fluorescent Double Staining

Fluorescent double staining was conducted to observe the apoptotic and necrotic morphological changes induced by levosimendan. C33A cells were seeded into 6-well plates with a density of 200,000 cells/well. After overnight incubation at 37 °C under 5% CO_2_, cells were treated with different concentrations of levosimendan for 24 h and 48 h. Cells were then stained with a medium containing lipophilic Hoechst 33258 (5 μg/mL, HO) and hydrophilic propidium iodide (3 μg/mL, PI) for 90 min under cell culture conditions. After the medium was substituted in the wells, images were taken with a Nikon Eclipse TS100 fluorescence microscope (Nikon Instruments Europe, Amstelveen, The Netherlands) equipped with appropriate optical filters for HO and PI. This procedure allows detection of early apoptosis and late apoptosis/necrosis by visualization of cellular nuclei morphology and membrane integrity. 

### 2.5. Cell Cycle Analysis

Flow cytometric analysis was performed to measure cellular DNA content. C33A cells were seeded into 12-well plates at a density of 100,000–150,000 cells/well. After overnight incubation, the samples were treated with escalating concentrations of the test compound for 24 and 48 h. The samples were collected for each well separately after a washing step with phosphate-buffered saline (PBS) and the trypsinization process, and the harvested cells and supernatants were centrifuged at 1400 rpm for 5 min at room temperature. After a washing step with PBS, cells were fixed in 300 µL of 70% ice-cold ethanol for 30 min. Cells were resuspended in a PI solution containing 10 µg/mL PI, 10 µg/mL RNase A, 0.1% Triton-X, and 0.1% sodium citrate solubilized in distilled water, and the samples were stored for 30 min in the dark, at room temperature. Cells were analyzed using a CytoFLEX-V0-B4-RO flow cytometer (Beckman Coulter, Brea, CA, USA). In each analysis, 20,000 events were measured, and the recorded data were evaluated by using ModFit LT 3.3.11 software (Verity Software House, Topsham, ME, USA) to determine percentages of the cells in each cell-cycle phase (subG1, G1, S, and G2/M). Untreated cells were considered as controls. Apoptotic cells were identified by sub-G1 phase in the cell-cycle distribution [52].

### 2.6. Wound-Healing Assay

A wound-healing assay was conducted to determine the levosimendan effect on cell motility using a special silicone insert (ibidi GmbH, Gräfelfing, Germany) embedded in a 12-well plate. C33A cells were seeded in both insert chambers at a density of 45,000 cells, and then the cells were incubated for 48 h to form a confluent monolayer. Next, the culture insert was removed, and a washing step was performed to remove the detached cells and debris. Lastly, cells were treated with increasing concentrations of the tested compound in a medium supplemented with 1% FBS. The ImageJ software (National Institutes of Health, Bethesda, MD, USA) determined the size of cell-free zones based on images taken with a Nikon Eclipse TS100 microscope at 0, 24, and 48 h post-treatment. The percentage of cell migration for treated samples was compared with control samples assessed at the same intervals. The migration assay was repeated twice with three parallel samples.

### 2.7. Boyden Chamber Assay

Boyden-chamber assay was carried out to identify treatment effects on C33A cells invasion capacity using the BD BioCoat Matrigel Invasion Chamber with an 8 µm-pore-size PET membrane with a thin layer of Matrigel Basement Matrix (BD Biosciences, Bedford, MA, USA). After at least two hours of prehydration, 500 µL suspension of C33A cells, prepared in serum-free MEM supplemented with different concentrations of levosimendan, were located into the upper chamber, while the MEM supplemented with 20% FBS was served as a chemoattractant in the lower chamber. After 48 h of incubation at 37 °C under 5% CO_2_, the supernatants and non-invading cells were mechanically eliminated with a cotton swab, and the chamber membranes were washed with PBS twice. The invaded cells were fixed in 96% ice-cold ethanol and stained with a 1% crystal violet solution in the dark for 30 min, at room temperature. A Nikon Eclipse TS100 microscope was used to capture at least 6 images per insert, and invading cells were counted. Untreated samples were considered as controls. Each condition was prepared in two parallel Boyden-chamber inserts with two time repetitions.

### 2.8. Statistical Analysis

Data are displayed as mean ± SEM from at least two separate experiments. Statistical analysis was performed using one-way analysis of variance (ANOVA) followed by Dunnett’s test to identify statistical significance using GraphPad Prism v 9.0 software for Windows (GraphPad Software, San Diego, CA, USA). *, **, and *** indicate *p* < 0.05, *p* < 0.01, and *p* < 0.001 compared to the control, respectively.

## 3. Results

### 3.1. Antiproliferative MTT Assay

Table 1 demonstrates the growth-inhibitory effects of levosimendan on the three tested cervical carcinoma cell lines. The results show that levosimendan exerted a higher growth-inhibitory effect on C33A cells with a 58.42 µM IC_50_ value with no substantial inhibitory potential on the SiHa and HeLa cells 72 h post-treatment (dose-response curves are presented in the Supplementary Materials (Figure S1)). The obtained IC_50_ values showed at least one order of magnitude lower levosimendan activity than the reference agent cisplatin. Non-cancerous fibroblast cells (MRC-5) were also used to characterize the tumor selectivity of levosimendan. Our data revealed that levosimendan is more selective towards C33A cells. Therefore, the C33A cell line was selected for further in vitro investigations.

### 3.2. Hoechst 33258–Propidium Iodide Fluorescent Double Staining

**Figure 1:**
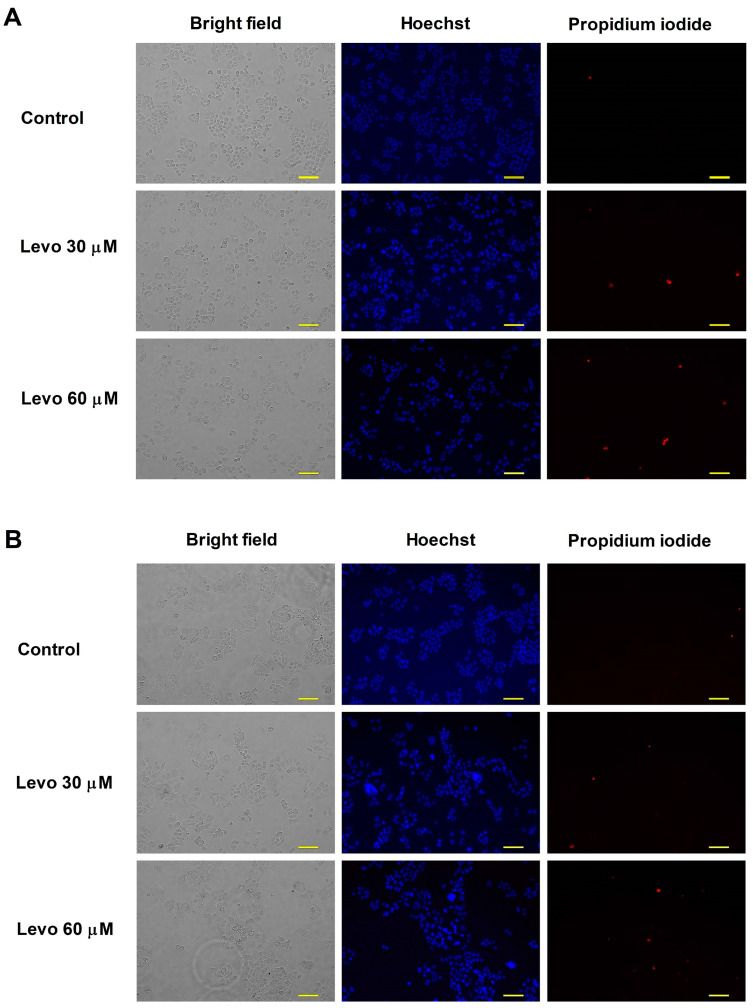
Changes in cell morphology and membrane integrity of C33A cells after 24 h (**A**) and 48 h (**B**) of levosimendan (Levo) treatment, visualized by HOPI fluorescence double staining; intense blue fluorescence indicates DNA fragmentation and nuclear blebbing referring to early apoptotic cells and red fluorescence shows secondary necrotic cells. The bar in the pictures indicates 100 µm.

To visualize the changes in the cell morphological appearance and membrane integrity of the C33A cells induced by the levosimendan, fluorescent nuclei double staining was performed using Hoechst 33258 and propidium iodide dyes 24 h and 48 h post levosimendan exposure. Images from the same field at the previously mentioned time interval were taken using different filters. Our results revealed early apoptotic cells with bright blue fluorescence due to DNA condensation. In contrast, the secondary necrotic cells emitted red fluorescence, indicating potentially damaged cell membranes and subsequent propidium iodide dye cellular uptake (Figure 1). The increase in apoptotic and necrotic cell populations presented concentration dependency relative to the untreated controls.

### 3.3. Cell Cycle Analysis

**Figure 2:**
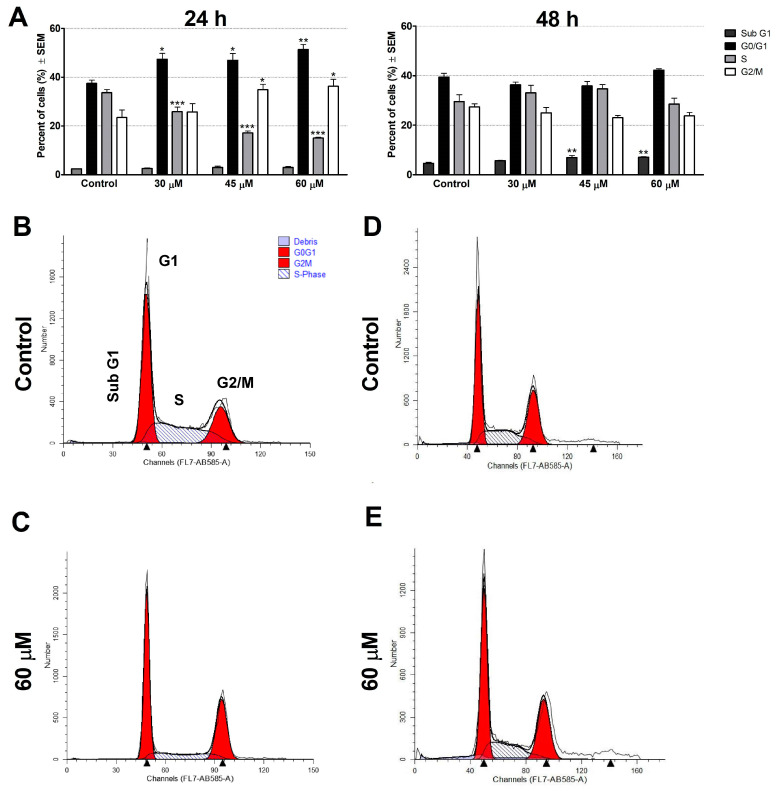
Cell cycle analysis by flow cytometry. Levosimendan-induced cell cycle disturbances, characterized by an increased rate of the G1 and G2/M cell populations at the expense of the S phase in C33A cells (**A**). Data are presented as mean ± SEM. *, **, and *** indicate *p* < 0.05, *p* < 0.01, and *p* < 0.001, respectively, compared to controls. Representative histograms: Controls (**B**,**D**) 24 h and 48 h post-treatment, respectively, and treated cells (**C**,**E**) 24 h and 48 h post-treatment, respectively. Findings are based on the results of two independent experiments done in triplicate.

The effects of levosimendan on the cell cycle were investigated by flow cytometry after 24 h and 48 h of incubation (Figure 2). The percentages of the cells in various cell cycle phases (subG1, G1, S, and G2/M) were determined. Levosimendan was found to induce cell-cycle disturbances at concentrations of 30 µM, 45 µM, and 60 µM, characterized by a significant accumulation of cells in the G1 phase accompanied by a concentration-dependent decrease in the proportion of cells in the S phase, with a modest accumulation of cells in the G2/M phase 24 h post-incubation. After 48 h of incubation, levosimendan resulted in a gradual accumulation of hypodiploid subG1 cell populations, a marker of induced apoptosis. These results suggest that levosimendan induces marked cell cycle disturbances 24 h post-treatment. After prolonged incubation, these changes appeared as accumulated cells at the apoptotic subG1 phase. Representative histograms of higher resolution are available in the Supplementary Materials (Figure S2).

### 3.4. Wound Healing

**Figure 3:**
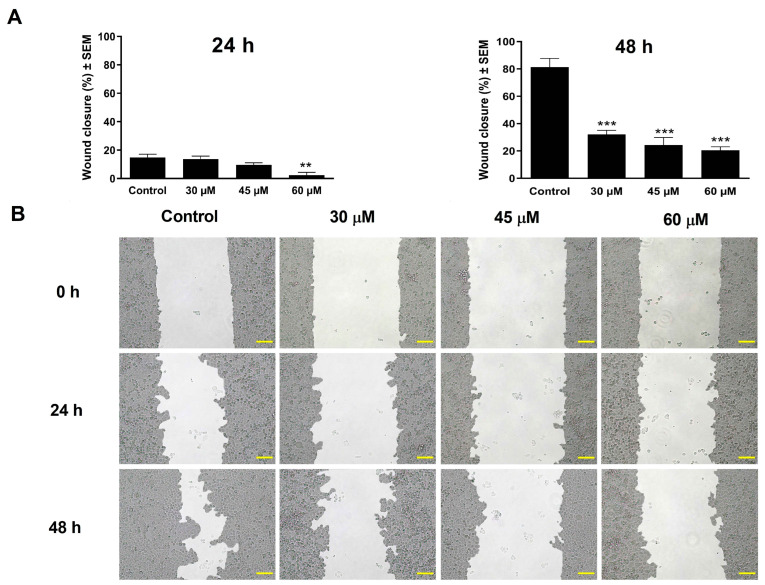
Effect of levosimendan on the migration of C33A cells (**A**). Graphs indicate the percentage of cell-free areas at 24 h and 48 h post-treatment in the C33A cell line compared to the controls (**B**). Representative images of reduced wound healing at 0, 24, and 48 h post-treatment. The bar in the pictures indicates 100 µm. Data are presented as mean ± SEM. ** and *** indicate *p* < 0.01 and *p* < 0.001, respectively, compared to controls. Findings are based on the results of two independent experiments done in triplicate.

In addition to inhibiting cell proliferation, levosimendan suppressed the migration of the cervical cell line C33A, evaluated by a wound-healing assay through determining image-derived cell-free gaps at 0, 24, and 48 h post-treatment with various concentrations of levosimendan. After 24 h of incubation, only a concentration of 60 µM produced substantial effects. However, a significant inhibition of cell migration was detected after 48 h at all tested concentrations compared to untreated controls (Figure 3). These findings conclude that levosimendan significantly reduces the cell migration ability in a concentration- and time-dependent manner.

### 3.5. Boyden Chamber Assay

**Figure 4:**
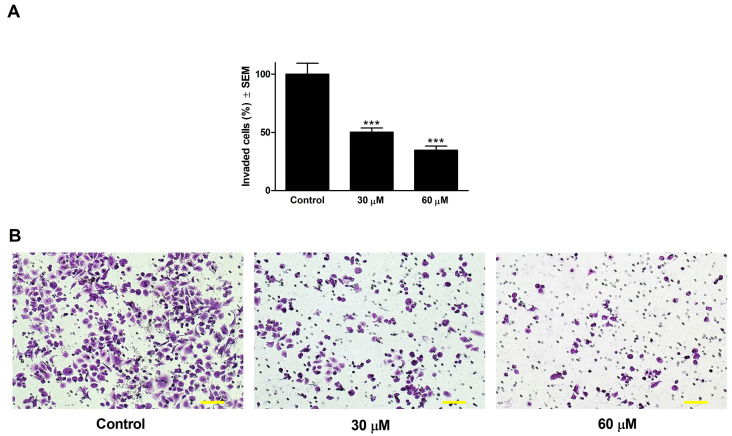
Effect of levosimendan on the invasion of C33A cells (**A**). The percentage of invading cells treated with levosimendan for 48 h (**B**). Representative images at 48 h post-treatment. The bar in the pictures indicates 100 µm. Data are presented as mean ± SEM. *** indicates *p* < 0.001, compared to untreated control samples. Findings are based on the results of two independent experiments done in duplicates.

To determine the anti-invasive capacity of levosimendan, a Boyden-chamber assay was performed, which simulates the extracellular environment of primary tumors in order to investigate cellular invasion. Quantitative analysis by counting the number of invaded cells in each well showed that levosimendan inhibited cell invasion in a dose-dependent manner compared with the untreated control 48 h post-treatment, even at sub-inhibitory concentrations (Figure 4).

## 4. Discussion

Cancer is a major health issue around the world, and it is expected that the global burden of cancer will increase. Although the limited success of current therapies has led to huge investments in drug development, the average number of FDA-approved drugs per year has declined since the 1990s [53,54]. This increasing need for more effective anticancer drugs has increased interest in drug repurposing. 

**Figure 5:**
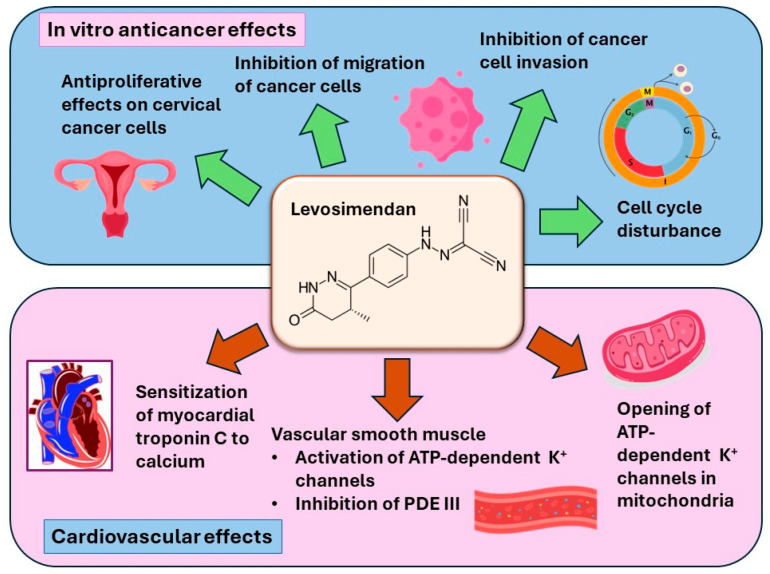
Potential pharmacological properties of levosimendan on cancerous cells in vitro and its cardiovascular effects.

Levosimendan is traditionally employed in cardiovascular management, especially in decompensated severe chronic heart failure [55]. Due to its diverse pharmacological properties, it has emerged as a candidate for repurposing in oncology (Figure 5). Additionally, knowledge of levosimendan’s safety profile increases the probability of its successful translation from preclinical research settings to clinical uses. Few previous studies have investigated the in vitro anticancer effects of levosimendan. A study evaluating the anticancer activity of levosimendan in several cancer cell lines of different origins revealed that lymphoma was the most sensitive to levosimendan [50]. Another study evaluated the combination of levosimendan with an antineoplastic drug, 5-fluorouracil (5-FU); this research found that the combination produced a pronounced synergistic effect in the treatment of bladder cancer cells [49]. On the other hand, many studies demonstrated that cardiac glycosides inhibit cell growth and induce apoptosis in numerous investigated cancer cell lines, including medulloblastoma, non-small lung cancer, renal adenocarcinoma, melanoma, breast adenocarcinoma, and leukemia. Most of these effects are potentially mediated through targeting Na^+^/K^+^ ATPase [56,57,58].

Our present study aimed to investigate the antineoplastic properties of levosimendan and to approach its possible mechanism of action. Antiproliferative assays were performed on three distinct cervical carcinoma cell lines with varying HPV statuses (Hela, SiHa, and C33A), and the IC_50_ values were calculated. Based on our results, the anticancer effect of levosimendan was more selective against HPV-negative C33A cells (IC_50_ values: 58.42 µM). However, the potency of levosimendan was found to be lower compared to a standard drug used in the routine therapy of cervical cancer cisplatin [59], which makes levosimendan suitable to be used as an adjuvant with standard care therapy to reduce tumor proliferation. In addition, levosimendan exhibited higher IC_50_ values in non-cancerous fibroblast cells, MRC-5, than in C33A cancer cells, indicating its tumor selectivity.

Furthermore, C33A cells underwent apoptosis after treatment with levosimendan based on the morphological changes visualized by fluorescent double staining and subG1 fractions resulting from DNA fragmentation after 48 h of incubation. Identification of the subG1 subpopulation may be used as an index of apoptotic cells in C33A cells treated with levosimendan.

Levosimendan activates nitric oxide (NO) production in endothelial cells by stimulating specific cellular pathways that involve key proteins: p38 mitogen-activated protein kinases (MAPKs), extracellular signal-regulated kinase (ERK), and protein kinase B (PKB/Akt) [48]. Numerous studies have shown the ability of NO to prevent cancer cell proliferation [60,61] and induce cell-cycle arrest [62]. In the current study, cell-cycle analysis was carried out using flow cytometry on C33A cells to explore the mechanism of action of levosimendan. Our results proved that levosimendan-induced cell-cycle disturbances were characterized by a considerable elevation of cells in the G1 phase and a reduction in the proportion of cells in the S phase, with a modest accumulation of cells in the G2/M phase. Moreover, the proportion of the hypodiploid subG1 fraction, regarded as the apoptotic population, was markedly elevated after 48 h of incubation.

The metastatic dissemination of cancer cells is the leading cause of cancer-related mortality. The invasion-metastasis cascade is a complex sequential and interrelated step leading to the formation of distant secondary tumors [63]. Earlier studies have reported that increasing the levels of cAMP in cancer cells can inhibit their in vitro migration [64,65]. Consequently, drugs that efficiently increase cAMP levels in cancer cells can potentially reduce or inhibit metastasis in cancer patients. Levosimendan and its active metabolite, OR-1896, are known to be highly selective PDE III inhibitors. This enzyme is responsible for the breakdown of cAMP [66]. In the current study, levosimendan significantly reduced the migration of C33A cells after 24 h of exposure only at the growth-inhibitory concentration. After 48 h of incubation, a pronounced difference, >40%, in the cell migration rate of control and treated samples was detected even at concentrations with minimal or no substantial antiproliferative activity. Further, levosimendan remarkably reduced the invasion ability of the C33A cell line in a concentration-dependent manner 48 h post-treatment. These findings suggest that levosimendan may have promising antimetastatic properties.

## 5. Conclusions

In conclusion, the present study demonstrated that levosimendan induced cell growth inhibition and apoptosis in HPV-negative human cervical cancer C33A cells. Levosimendan altered the cell-cycle distribution by increasing the proportion of cells in the G1 and G2/M phases, decreasing the proportion of the cells in the S phase, and increasing the pro-apoptotic subG1 phase in a concentration-dependent manner, suggesting a potential mechanism of action underlying its antitumor effects on the C33A cell line. Furthermore, it effectively inhibits the migration and invasion of C33A cells in a dose-dependent manner. Based on these findings, the repurposing of levosimendan in the adjuvant settings might be considered to contribute to the antitumor and antimetastatic effects of ongoing HPV-negative cervical cancer chemotherapeutic regimens, as well as to reduce the risk of cardiotoxic side effects that are associated with several anticancer therapies [67]. However, many aspects still need to be investigated and clarified before the clinical use of a well-known drug in a new indication. 

## Figures and Tables

**Table 1:** Antiproliferative properties of levosimendan on human cervical cancer cell lines and a non-cancerous fibroblast cell line (MRC-5).

Cell Lines	Levosimendan IC_50_ (µM) [95% CI (µM)]	Cisplatin IC_50_ (µM) [95% CI (µM)]
SiHa	128.80 [109.30–148.20]	4.29 [3.72–4.95]
HeLa	170.00 [144.50–195.50]	12.14 [10.18–14.46]
C33A	58.42 [45.75–71.08]	5.85 [5.37–6.38]
MRC-5	315.70 [304.70–326.70]	4.96 [3.51–6.99]

CI; confidence interval.

## Data Availability

The data of this study can be made available on a reasonable request basis. This request must be addressed to the correspondence author of the research group István Zupkó (zupko.istvan@szte.hu).

## Supplementary Materials

The following supporting information can be downloaded at: https://www.mdpi.com/article/10.3390/cimb46070391/s1, Figure S1: Growth inhibitory effects of levosimendan on cervical cancer cell lines and non-cancerous human fibroblast cells (MRC-5); Figure S2: Representative histograms for C33A cervical cell line treated with levosimendan.
