# Supplementary material for: An In Vitro Investigation of the Antiproliferative and Antimetastatic Effects of Levosimendan: Potential Drug Repurposing for Cervical Cancer
Source: Curr Issues Mol Biol. 2024 Jun 27;46(7):6566–79. doi: 10.3390/cimb46070391 (PMC11275392; doi:10.3390/cimb46070391)

Supplementary materials

for

An in vitro investigation of the antiproliferative and antimetastatic effects of levosimendan: Potential drug repurposing for cervical cancer

Zsuzsanna Schelz [†], Hiba F. Muddather [†], Fatemeh Sheihaki Jaski, Noémi Bózsity and István Zupkó *

Institute of Pharmacodynamics and Biopharmacy, Faculty of Pharmacy, University of Szeged, Eötvös u. 6, H-6720 Szeged, Hungary; schelz.zsuzsanna@szte.hu (Z.S.); hiba.161991@hotmail.com (H.F.M.); jaski.sheihaki.fatemeh@gmail.com (F.S.J.); bozsity-farago.noemi@szte.hu (N.B.)

* Correspondence: zupko.istvan@szte.hu

† These authors contributed equally to this work.

Figure S1. Growth inhibitory effects of levosimendan on cervical cancer cell lines and non-cancerous human fibroblast cells (MRC-5):

A. **First experiment:**

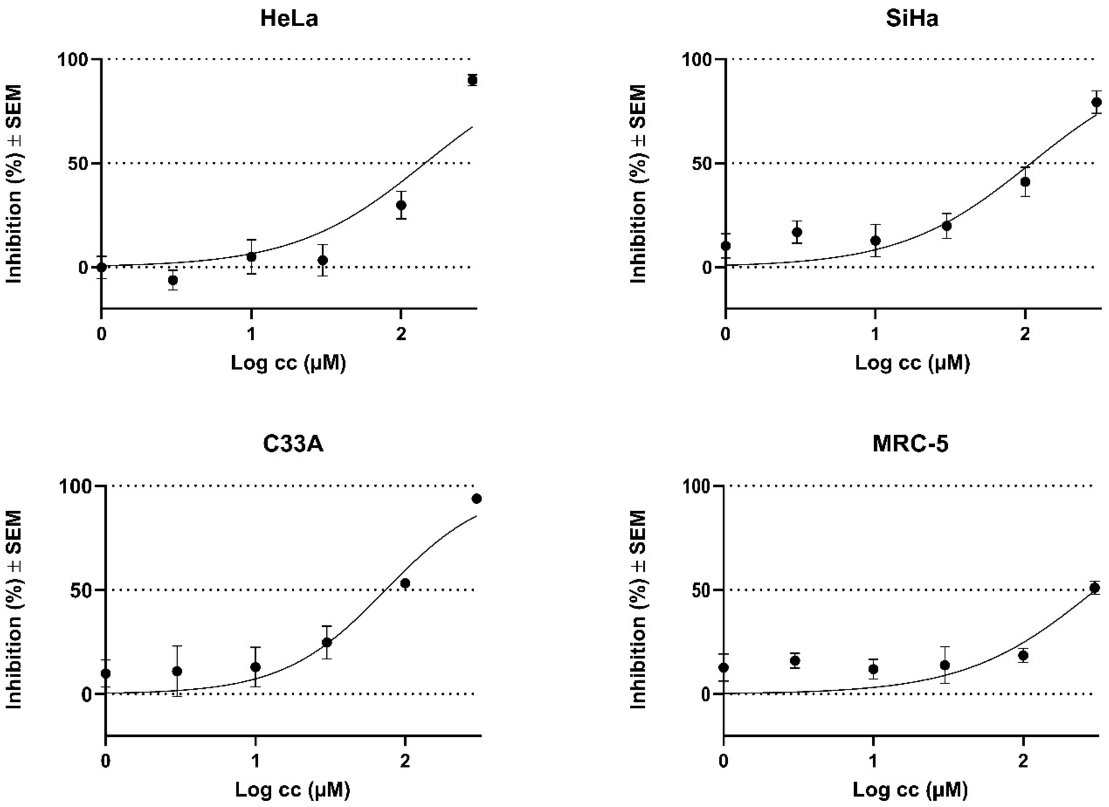

Data presents dose-response curves as the average percentages of cell growth inhibition ± SEM on C33A cells, 72 h post-incubation, measured by a standard MTT assay.

B. **Second independent experiment:**

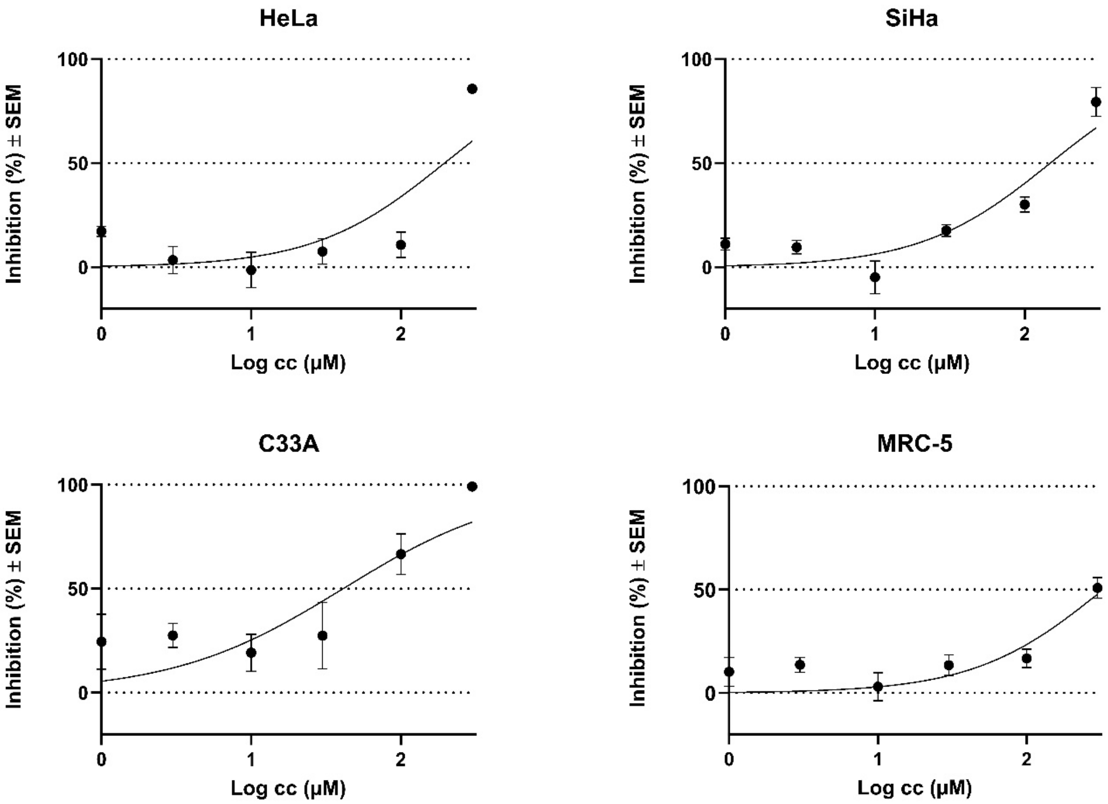

Data presents dose-response curves as the average percentages of cell growth inhibition ± SEM on C33A cells, 72 h post-incubation, measured by a standard MTT assay.

Figure S2. Representative histograms for C33A cervical cell line treated with levosimendan:

A. 24 h post-incubation:

Control

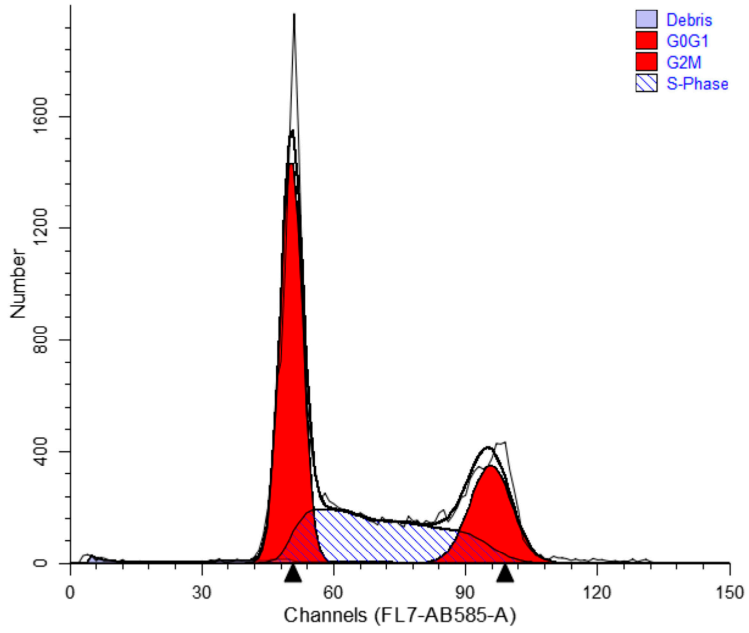

30 μM levosimendan

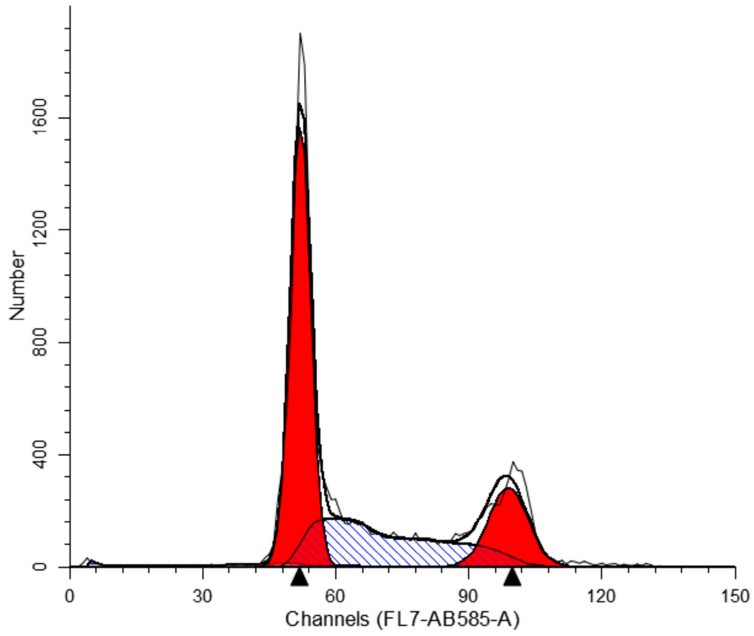

45 μM levosimendan

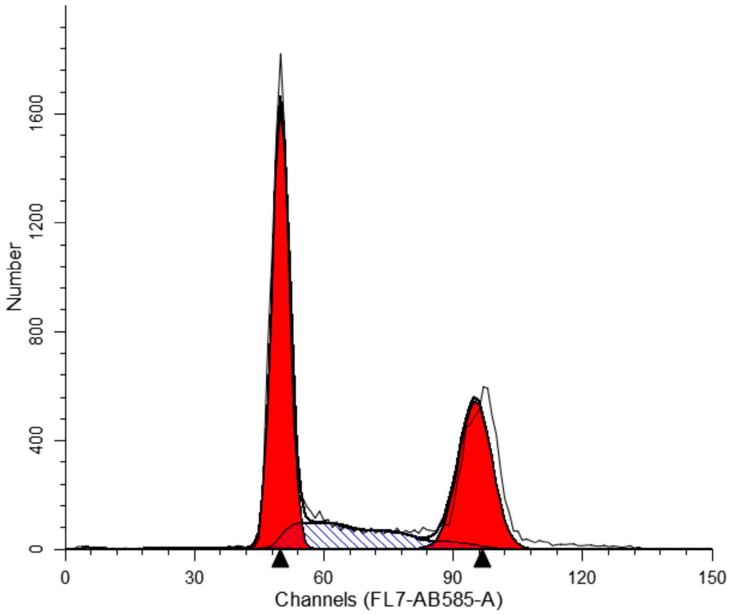

60 μM levosimendan

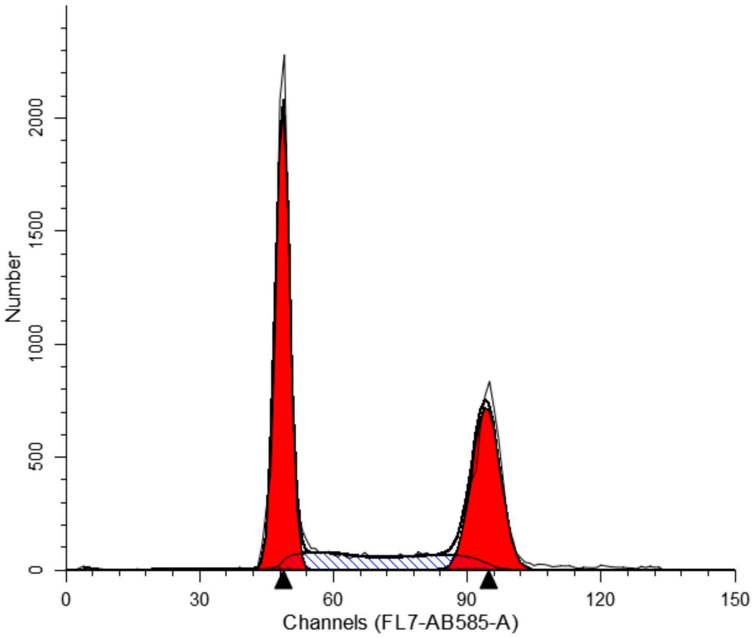

B. 48 h post-incubation:

Control

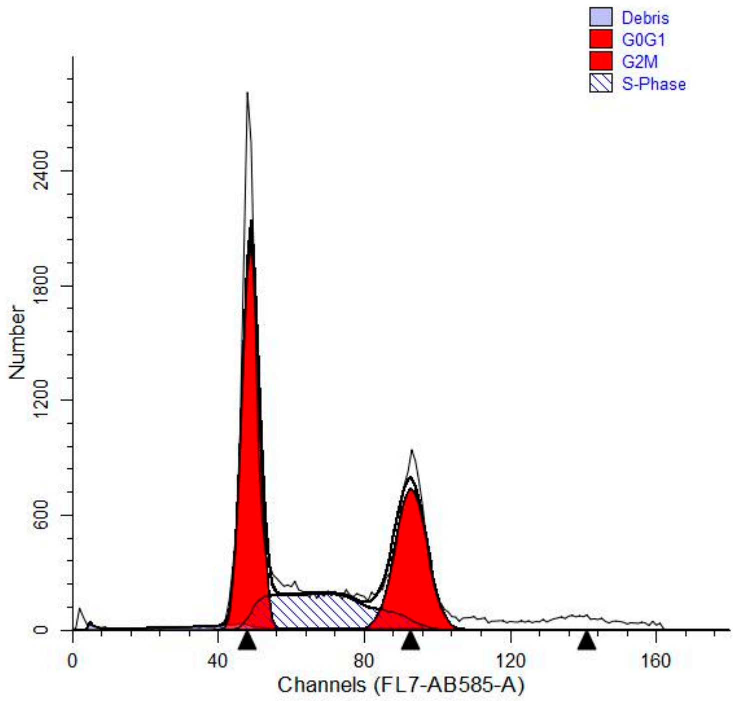

30 μM levosimendan

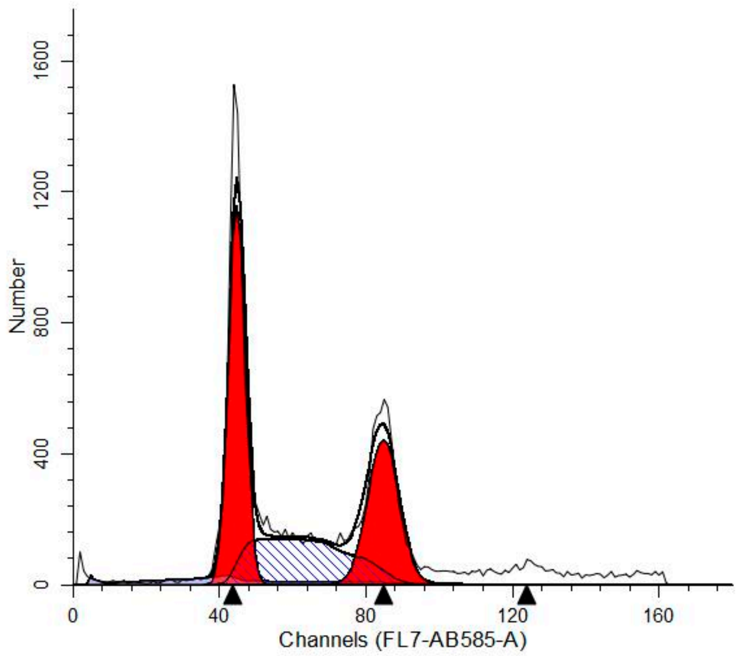

45 μM levosimendan

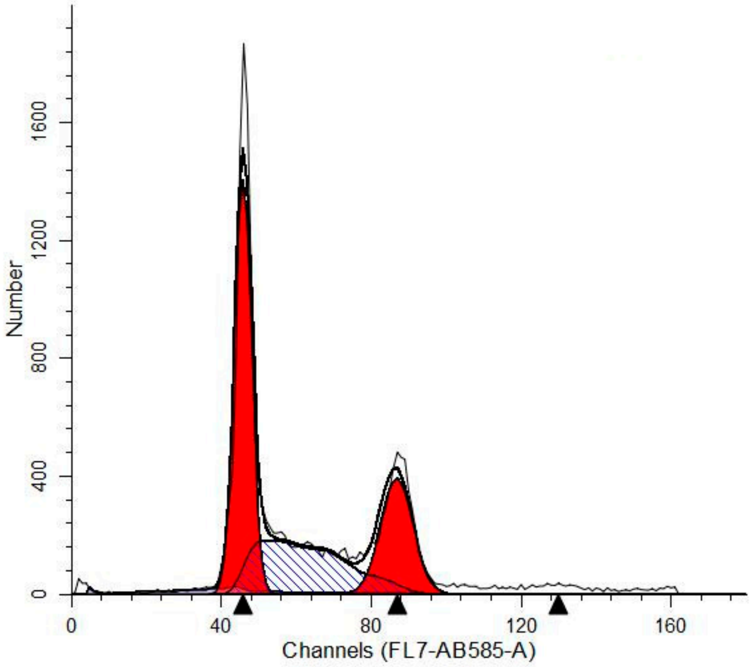

60 μM levosimendan

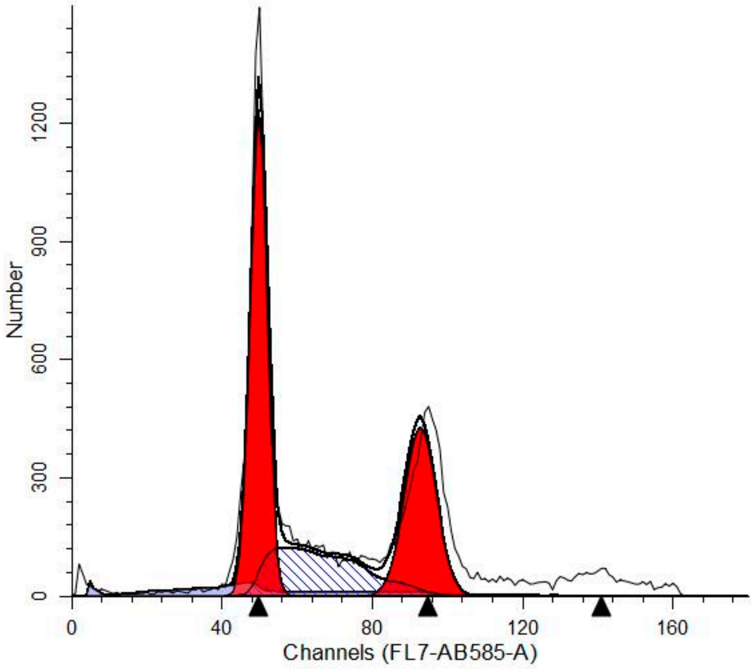

Supplement: Supplementary file 1 [file cimb-46-00391-s001.zip › cimb-3061493-supplementary.pdf]
